# Rising Oceans Guaranteed: Arctic Land Ice Loss and Sea Level Rise

**DOI:** 10.1007/s40641-018-0107-0

**Published:** 2018-07-10

**Authors:** Twila Moon, Andreas Ahlstrøm, Heiko Goelzer, William Lipscomb, Sophie Nowicki

**Affiliations:** 1 National Snow and Ice Data Center (NSIDC), Cooperative Institute for Research in Environmental Sciences (CIRES), 449 UCB, University of Colorado, Boulder, CO 80309-0449 USA; 2 Department of Glaciology and Climate, Geological Survey of Denmark and Greenland, Copenhagen, Denmark; 3 Institute for Marine and Atmospheric Research Utrecht (IMAU), Utrecht University, Princetonplein 5, 3584 CC Utrecht, The Netherlands; 4 Laboratoire de Glaciologie, Université Libre de Bruxelles, Brussels, Belgium; 5 National Center for Atmospheric Research (NCAR), Boulder, CO USA; 6 NASA Goddard Space Flight Center, Greenbelt, MD 20771 USA

**Keywords:** Arctic, Sea level rise, Glaciers, Ice caps, Remote sensing, Greenland Ice Sheet

## Abstract

**Purpose of Review:**

This paper reviews sea level contributions from land ice across the Arctic, including Greenland. We summarize ice loss measurement methods, ice loss mechanisms, and recent observations and projections, and highlight research advances over the last 3–5 years and remaining scientific challenges.

**Recent Findings:**

Mass loss across the Arctic began to accelerate during the late twentieth century, with projections of continued loss across all future greenhouse gas emission scenarios. Recent research has improved knowledge of ice hydrology and surface processes, influences of atmospheric and oceanic changes on land ice, and boundary conditions such as subglacial topography. New computer models can also more accurately simulate glacier and ice sheet evolution.

**Summary:**

Rapid Arctic ice loss is underway, and future ice loss and sea level rise are guaranteed. Research continues to better understand and model physical processes and to improve projections of ice loss rates, especially after 2050.

## Introduction

The Arctic contains 3.1 × 10^6^ km^3^ of land ice, equivalent to 7.7 m of sea level rise [1••, 2••], of which about 97% is stored in the Greenland Ice Sheet (GrIS). Land ice loss is accelerating due to rapid system-wide changes in Arctic climate, predominantly caused by human greenhouse gas emissions. Across the Arctic, temperatures are rising much faster than the global mean; during 1880–2012, the Arctic warmed ~ 3.5 °C compared to 0.85 °C for the Earth overall (1880–2012) [3, 4••]. Arctic climate change is causing variations in ocean temperature and changing atmospheric and cloud conditions. These changes influence land ice and, together, are melting substantial amounts of ice and raising sea levels across the globe. In this brief review, we focus on the scientific results and advances over the last 3–5 years, remaining challenges for understanding land ice loss from the Arctic, and the implications for sea level rise.

Land ice is a single component of the larger sea level rise budget, but an increasingly important one [5•, 6]. Current global average sea level rise is the combined result of steric changes caused by thermal expansion of ocean water and barystatic changes caused by the melting of land ice and a relatively small contribution from changes in land water storage. The primary influence of land ice loss is an increase in ocean water mass as solid ice mass is removed from land and added to the ocean. Many studies of total sea level change use ocean surface height or Earth gravity field measurements to examine sea level rise as a whole [6, 7•, 8], while others use tide-gauge measurements of relative sea level rise [7•]. Meanwhile, some research seeks to understand the separate components contributing to sea level rise [5•, 6]. Parsing the different contributors helps us to understand the rates of past, present, and future sea level rise, as well as how local and regional sea level rise will differ from the global average due to factors such as the location of ice loss, differences in land subsidence, and variations in oceanic and atmospheric circulation. Arctic land ice loss is and will continue to be a primary contributor to sea level rise. Other ice-covered regions, especially the Antarctic Ice Sheet, are also making a growing contribution to sea level rise but are not considered in this paper.

### Measuring Land Ice Change

Measuring mass change in glaciers and ice sheets is not straightforward. A variety of techniques are well established, however, and measurements of Arctic ice loss are in good agreement [9••, 10, 11••]. The advent of satellite monitoring in the 1990s was a major advance for studying large ice areas, and our ability to estimate ice mass loss has correspondingly improved in the beginning of the twenty-first century. Long-term in situ monitoring projects and paleo-glaciological studies have helped to extend the historical record and provide upper and lower limits on sea level rise from past land ice loss. Below, we briefly outline the techniques most commonly used to determine mass balance. All have advantages and disadvantages, discussed in more detail elsewhere [9••, 11••, 12]. Combined results, however, give a clear picture of accelerating Arctic ice loss during the recent one to two decades [9••, 11••].

#### Gravity Measurements

Measurements of Earth’s changing gravity field via the Gravity Recovery and Climate Experiment (GRACE) satellite mission (2002–2017) and GRACE Follow On (GRACE-FO, launched May 2018) are used to estimate ice mass variations. While separating ocean mass change from land ice mass change and correcting for vertical land motion requires careful processing, gravity measurements produce high-quality data on ice mass changes, especially for large ice areas like Greenland [13–15]. With a limited ability to measure small (< ~ 300 km) regions, gravity measurements integrate regional mass change, for example covering the northern Canadian High Arctic as one region and combining Greenland peripheral glaciers and ice caps with the main ice sheet.

#### Volume to Mass via Ice Elevation

Repeat altimetry and digital elevation model (DEM) differencing allow measurements of volume change, which is then converted to mass change (with assumptions about snow, firn, and ice density). Laser and radar altimetry data from airborne (e.g., Operation IceBridge, PROMICE) and satellite (e.g., ICESat, Cryosat2) platforms is useful for small and large ice areas [16–20]. It can be difficult, however, to retrieve altimetry measurements over rugged, steeply sloped ice sheet margins, and processing must account for radar penetration into the surface snow or firn. Relying on remote sensing instruments, these records are concentrated within the first two decades of the twenty-first century.

#### Input-Output or Mass Budget Method

The mass change of an ice body is the difference between the input (mainly snow accumulation through precipitation) and the output (surface melting and runoff, along with the discharge of solid ice to the ocean). Scientists calculate this mass budget by combining data or modeling of the surface mass balance (SMB)—the difference between accumulation and ablation at the ice surface—with information about ice dynamics, such as iceberg calving. (Meltwater that refreezes in the snowpack is not part of the SMB, since it does not alter the mass.) The result is not a measure of total mass but of mass change over some period (usually on annual or longer time scales) [21•, 22, 23].

#### Individual Glacier Observations

Changes in length, volume, and mass have been monitored routinely for a small number of individual glaciers, some for more than a century. Those glaciological data (e.g., in situ stake measurements) and geodetic observations have been compiled by the World Glacier Monitoring Service (WGMS) for several hundred glaciers, including many in the Arctic. Although labor-intensive and limited in spatial coverage (hence not ideal for ice sheets), these measurements provide a high-fidelity record of glacier mass balance changes from the early twentieth century through the present [24•].

Another technique applied to glaciers and ice caps is volume-area scaling [25]. Based on dimensional analysis, glacier volume can be estimated from area and length, which are easier to measure. While there are large uncertainties in converting area and length changes to mass changes, this method can take advantage of multi-decadal records for hundreds of the Earth’s glaciers.

#### Modeling

Sophisticated dynamical ice sheet models are used to estimate future Greenland mass changes resulting from modified SMB and ice flow [26, 27, 28•, 29, 30]. These models can be run either in standalone mode with prescribed climate forcing [31•], or within coupled climate models [32•, 33]. The models are validated by comparison to observed ice sheet area, thickness, and velocity, and are starting to be used to hindcast recent changes [34•]. Computer models can also simulate aggregated mass losses from the Earth’s glaciers [35]. Compared to ice sheet models, global glacier models typically have simplified dynamics, focusing on SMB changes or process understanding.

### Mechanisms of Land Ice Loss

The warming atmosphere and ocean are the foundational cause for accelerating Arctic ice loss, but it is useful to consider the specific mechanisms driving ice loss. Understanding these mechanisms is particularly important for the challenge of understanding ice loss *rates*. Ice loss fits into two broad categories: loss due to changes in SMB and loss due to iceberg calving, often called dynamic ice loss. SMB changes arise from variations in solar and longwave radiation, air temperature, and the amount and phase of precipitation (rain or snow). Other surface processes, like refreezing of surface melt water in the glacier firn [36•] or increased surface melt due to a lower albedo (e.g., from algae growth [37] or changes in ice crystal size [38]), also affect SMB.

Solid ice discharge requires a marine connection. The rate of ice discharge is determined by the speed of glacier motion and the advance or retreat of the glacier ice front (a.k.a., terminus). Ice front stability can be influenced by ocean temperature and melt at the terminus and by glacier thinning from surface or bottom melt [39•, 40]. Topography and buttressing are also primary controls on the progression and rate of ice loss for marine-terminating glaciers [41•, 42]. Retrograde slopes that become deeper up-glacier increase retreat and ice loss, while buttressing from floating ice can enhance stability, and even small-scale topographic highs can act as pinning points [43, 44•]. Vertical fjord geometry also plays a role, with narrow fjord areas stabilizing glacier position [42].

For the GrIS and other large marine-connected ice caps and glaciers, the timescales and range of variability for SMB and dynamic discharge are different. SMB has higher year-to-year variability than solid ice discharge because it can respond quickly to atmospheric changes. For example, record GrIS melt years with exceptional mass loss in 2010 and 2012 helped to shift the SMB-to-discharge ratio from roughly 50:50 during 2000–2005 to 68:32 during 2009–2012 [23]. Due to increased ablation and limits on increasing ice discharge, the future contribution of SMB changes relative to solid ice discharge is expected to increase across the Arctic [30, 32•, 45, 46].

### Recent Arctic Land Ice Loss

**Fig. 1:**
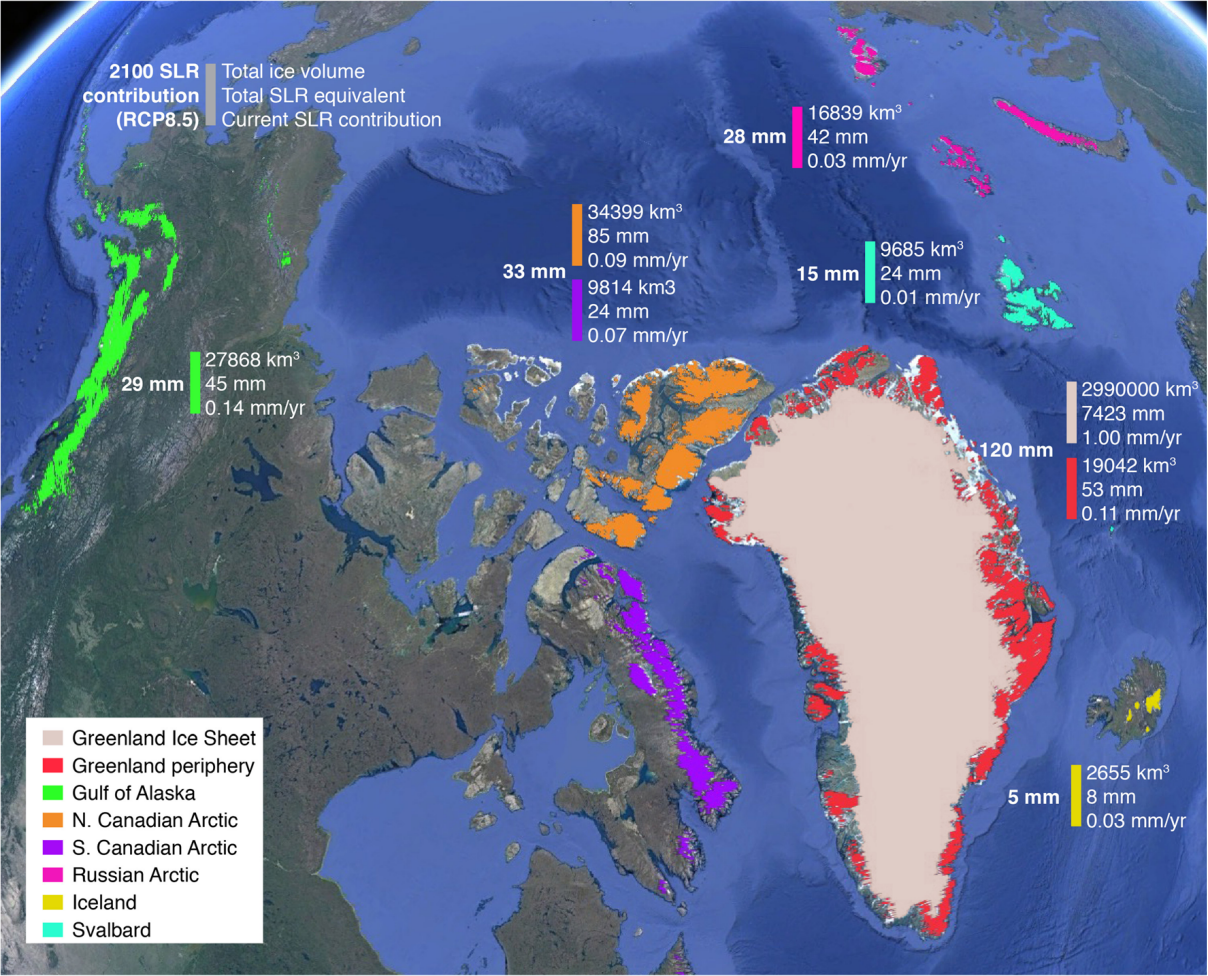
Location of Greenland and Arctic land ice. Values shown for total ice volume (km^3^), equivalent sea level rise potential (mm), current (2003–2009) sea level rise contribution (mm/year), and projected contribution to sea level rise during 2006–2100 (mm) for the RCP8.5 emissions scenario [1••, 4••, 35, 47•, 63]

The observational record of widespread Arctic (and global) land ice loss over the last two to three decades is well established, with significant contributions to global sea level rise. The magnitude of ice loss varies across the Arctic. Current GrIS contributions are roughly double those from other Arctic glaciers and ice caps. The ranking of recent (~2003–2009) Arctic mass loss from most to least is GrIS alone (~ 360 Gt/year), Alaska (~ 50 Gt/year), Greenland peripheral glaciers and ice caps (~ 38 Gt/year), North Canadian Arctic (~ 33 Gt/year), South Canadian Arctic (~ 27 Gt/year), Russian High Arctic and Iceland (~ 11 Gt/year each), and Svalbard (~ 5 Gt/year) [1••, 47•, 48, 49] (Fig. 1).

Ice loss from Greenland accelerated over the beginning of the twenty-first century and will continue to be the largest source of Arctic land ice loss. The ice sheet was likely close to a balanced state during the 1970s and 1980s but transitioned into negative mass balance beginning in the early to mid 1990s [11••, 50]. Ice loss during 2003–2013 averaged 280 ± 58 Gt/year [14], which is consistent with a wide range of mass loss studies [11••]. This decadal average, however, masks the more important point: Greenland ice loss has accelerated rapidly. Acceleration during 2003–2013 was 25 ± 1.2 Gt/year^2^ [14], and there is no indication that this trend will stop. Ice loss during 2009–2012, for example, was ~ 360–380 Gt/year [23, 48]. These losses translate into a mean 2003–2013 sea level rise contribution of 0.8 ± 0.2 mm/year. Contributions during the record year of 2012 reached as high as 1.2 ± 0.3 mm/year [50]. Since 2000, all sectors of the ice sheet have lost ice [11••], though the southern portion of the GrIS is more sensitive to climate warming and may be further out of balance than the northern portion during coming decades [51].

As Greenland transitioned to negative mass balance, changes in SMB and solid ice discharge contributed roughly similar amounts to mass loss [10, 23]. Record melt years in 2010 and 2012, however, skewed this balance towards greater contributions from SMB [23], with an expectation that this gap will widen in the future [30, 32•, 45, 46]. The spatial pattern of ice loss and gain also varies across Greenland. Remote sensing data from the ice sheet interior suggest mass gain, with a 2007–2011 average additional mass accumulation of 41 ± 61 Gt/year for regions ≥ 1700 m above sea level [21•]. This increase, however, is more than offset by enhanced ablation and solid ice discharge at lower elevations.

Modes of climate variability, such as the Atlantic Multidecadal Oscillation (AMO), North Atlantic Oscillation (NAO), and atmospheric blocking events, all influence ice loss [52, 53, 54•]. For example, the NAO modulates regional Greenland precipitation, with increased east coast precipitation in a negative NAO phase and increased west coast precipitation in a positive NAO phase [55]. Similarly, increasing surface melt in southwestern Greenland has been tied to a recent atmospheric shift towards more frequent meridional exchange events during atmospheric blocking over Greenland [56]. Shifts in the NAO have also been linked to decreased Greenland cloud cover from 1995 to 2009, increasing surface melt [57], and to reduced glacier retreat in parts of the Russian High Arctic [58].

Natural climate variability was the primary driver in earlier Greenland ice loss. Records back to the nineteenth century show that there were periods of substantial Greenland glacier retreat and ice loss following the Little Ice Age (LIA; ending roughly 1890–1910) and during the 1930s–1940s warm period. However, glaciers at the start of the LIA were longer than today, reaching lower elevations and flatter terrain at their termini [59], and thus were sensitive to small temperature changes. Natural variability will also influence mass balance in the future, and we should not be surprised by years of little or no mass loss (e.g., during 2013 in Greenland [11••]). Because natural variability is now superimposed upon a dominant baseline trend of human-caused warming, however, there is no expectation of long-term (decadal or longer) cooling or opportunity for long-term glacier or ice sheet growth.

Arctic regions outside of Greenland are also losing substantial quantities of ice [9••]. Alaska is currently the second largest contributor to Arctic ice loss (trailing well behind the main GrIS). GRACE measurements provide a range of ice loss estimates, from 68.8 ± 11.0 Gt/year during 2003–2010 for the Gulf of Alaska total [60] to 36 ± 4 Gt/year in the northern Gulf of Alaska and 4 ± 3 Gt/year in the southern Gulf of Alaska during 2003–2013 [61]. Altimetry-based measurements during 1994–2013 give 75 ± 11 Gt/year ice loss [62] and 50 ± 17 Gt/year during 2003–2009 [47•], overall supporting the larger GRACE estimate. With 70% of Alaska ice loss attributable to land-terminating glaciers, it is clear that SMB is the primary cause of Alaskan ice mass loss [62].

The Canadian Arctic archipelago contains roughly one third of the Earth’s ice outside the AIS and GrIS [63]. GRACE estimates show mass losses of 72 ± 4 Gt/year during 2004–2011, with 2004–2006 losses at the lower rate of 31 ± 8 Gt/year compared to 92 ± 12 Gt/year for 2007–2009 [64]. Like Alaska, Canadian Arctic ice loss is dominated by surface melting, which contributed ~ 92% of the total ice loss during 2003–2009 [64]. Total mass loss has been somewhat greater in the North Canadian Arctic (38 ± 2 Gt/year) than the South (22 ± 2 Gt/year), with greater acceleration in the North during 2003–2013 [61]. GRACE measurements for Ellesmere and Devon Island indicate that ice loss was minimal during 2002–2008 but accelerated after 2008 [65••]. The Canadian Arctic currently contributes ~ 0.17 mm/year to rising seas [1••].

The Russian High Arctic contains roughly 20% of Arctic glacier ice outside of Greenland [1••], with glacier ice concentrated on the Novaya Zemlya, Severnaya Zemlya, and Franz Josef Land archipelagos. As with other Arctic regions, there was substantial ice loss and glacier retreat in the Russian Arctic during the first two decades of the twenty-first century as compared to the last quarter of the twentieth century (with earlier observations largely absent) [47•, 58, 66]. Ice loss estimates from the Russian High Arctic during 2003–2009 range from 9.1 Gt/year [66] to 11 Gt/year [47•], with other research finding a regional mass balance of − 6.9 ± 7.4 Gt/year during 2004–2012 [49]. More than 80% of Russian High Arctic ice loss during 2003–2009 occurred in Novaya Zemlya [47•, 66]. Currently, the Russian High Arctic contributes ~ 0.03 mm/year to sea level rise [1••].

The Vatnajökull Ice Cap contains ~ 80% of the glacial ice in Iceland, with other small glaciated areas also mostly in the southeast [67]. GRACE observations of Iceland show average losses of 10.9 ± 2.1 Gt/year during 2004–2012 (~ 0.03 mm/year of sea level rise), with a notable negative trend over the period [49]. This estimate compares well with altimetry-based calculations of 10 ± 2 Gt/year during 2003–2009 [47•]. The 2004–2012 average is a substantial increase from field-based estimates of 2.4 ± 2.2 Gt/year of ice loss during 1961–2003, though the extrapolation required for this calculation introduces substantial uncertainty [67].

Svalbard has had the smallest mass loss of the regions discussed here. Surface mass balance estimates over 2003–2009 give a loss of 5 ± 2 Gt/year [47•], and total mass balance estimates over 2004–2013 (including both ice front ablation and SMB) indicate losses of 13.4 Gt/year, corresponding to 0.037 mm/year of sea-level rise [68]. Using a climate reanalysis-forced model to determine Svalbard mass changes over 1957–2014 suggests that the current negative mass balance regime began in roughly 1980 [68]. Greater mass loss is projected in the future [63, 69, 70], which will reduce Svalbard’s 6700 ± 835 km^3^ of ice, equivalent to 17 ± 2 mm of potential sea level rise [71].

### Future Sea Level Rise

The Arctic will be a major source area for future sea level rise. Greenland is now a larger contributor than the world’s glaciers and ice caps (Antarctica currently trails both), while Arctic glaciers and ice caps (including Greenland’s periphery) are the largest combined source outside of the ice sheets [72].

Ice loss from the GrIS will continue to 2100 under all of the Intergovernmental Panel on Climate Change (IPCC) Representative Concentration Pathways (RCPs) used for projecting the climate response to varying additional levels of radiative forcing (e.g., RCP8.5 refers to 8.5 W/m^2^ additional radiative forcing in 2100 as compared to pre-industrial values) [4••]. In fact, ice loss is expected to continue for many centuries to millennia. Projections across different scenarios are fairly consistent for the next few decades but diverge substantially after ~ 2050 depending on greenhouse gas emissions [73••, 74]. Over decadal to centurial timescales, SMB will continue to dominate Greenland ice loss relative to ice discharge changes [33, 46], with SMB variability also expected to increase [32•, 45, 46]. Recent work projects that sea level rise contributions in 2100 from GrIS SMB will be 92 ± 45 mm under RCP8.5 forcing [51], similar to the likely range of the last IPCC assessment of 30–160 mm [4••]. The latter projects GrIS solid ice discharge contributions of 20–70 mm. One recent study (using a higher-order ice-dynamic model forced by 10 atmosphere and ocean general circulation models and four RCP scenarios) projects sea-level rise in the range from 42 ± 18 mm for RCP2.6 to 102 ± 32 mm for RCP8.5 from combined SMB and solid ice discharge changes [45].

Unconnected and weakly connected glaciers and ice caps in the GrIS periphery, typically assessed separately, are projected to contribute roughly 20 mm to sea-level rise under RCP8.5 [75]. Significant ice loss will also continue outside of Greenland. Projections for Gulf of Alaska glacier mass loss over 2010–2100 indicate sea level rise contributions of 14 ± 5 mm under RCP2.6 to 29 ± 6 mm under RCP8.5 [35]. Projections for Canadian Arctic glaciers and ice caps under RCP4.5 suggest that the region will contribute 33 mm to sea level rise by 2100, at a rate of 0.35 ± 0.24 mm/year [76], though other efforts suggest more modest contributions of ~ 20 mm [35]. Sea level rise due to Russian High Arctic ice loss, estimated by applying RCP4.5 and RCP8.5 emissions scenarios to 14 global climate models, is projected at 20–28 mm by 2100 [63]. Recent projections of Iceland contributions under RCP8.5 agree well at 4.7 ± 1.7 mm [35] and 4.9 mm [63]. For Svalbard, a study using the Modèle Atmosphérique Régional (MAR) regional climate model with RCP8.5 projects a rapid acceleration of surface melt near mid-century, with a negative surface mass balance over all glaciated areas by 2085 [69]. Projections for Svalbard mass loss during 2006–2100 using RCP4.5 are 12.4 mm sea-level equivalent (~ 55% of the total ice volume) [63], while estimated Svalbard mass loss under RCP8.5 forcing ranges from 14.0 to 16.4 mm [35, 63, 75]. Ice loss from glaciers and ice caps will continue after 2100, perhaps even without further warming. For example, recent computer simulations show that the Canadian Barnes Ice Cap, a remnant of the Laurentide Ice Sheet, will disappear within the next millennium under current climate conditions [77].

While we have presented estimates of the mean Arctic contributions to sea level rise, local sea level rise can be much higher or lower than the global mean. Local sea level rise is influenced by factors including land subsidence, ocean currents, atmospheric circulation, and the origin of ice loss. The loss of gravitational pull caused by ice mass loss, along with vertical land motion associated with unweighting, means that sea level can fall locally (within ~ 2000 km of the location of ice mass loss) while rising in more distant locations [78]. Fortunately, understanding of the zone of influence for ice mass loss has improved through a better understanding of where ice loss is expected and improved modeling of the resultant fingerprint of sea level rise [78, 79•]. For example, sea level rise in Los Angeles is more responsive to Greenland ice loss than other major US coastal cities and southeast Greenland ice loss is more important for sea level rise in Kodiak, AK, while northeast Greenland ice loss is more important for Miami, FL [79•]. As our abilities to project the location and rate of ice loss improve, that can inform and improve our understanding of where sea levels will rise most rapidly, and by how much, worldwide.

### Scientific Advances

Substantial research, particularly over the last one to two decades, has focused on understanding and projecting Arctic ice loss, leading to the various estimates outlined above. Earlier papers have discussed progress along the way [11••, 65••]. Here, we highlight significant advances over the last several years.

#### Glacier Change Is a Global Climate Signal

Evidence has been mounting that glaciers are declining worldwide, with the few exceptions attributed to local differences in climate change response (e.g., increased precipitation). Recent statistical analysis confirms that local glacier retreat (occurring globally) provides categorical evidence of climate change [80•, 81]. Quantifying the link between anthropogenic climate change and land ice loss supports the use of glacier and ice sheet retreat, and associated sea level rise, as a visually powerful example of the impacts of increasing greenhouse gas concentrations.

#### Arctic Records Extended Back into Early 1900s

The limited length of observational records continues to pose a challenge for assessing long-term trends, particularly before the satellite era. Data recovery from old photos, application of field techniques to map previous ice extents, and model hindcasts are helping to fill out the record [24•, 82–84]. Analysis reaching back to 1900, for example, indicates that Greenland mass loss rose substantially during 2003–2010 (186.4 ± 18.9 Gt/year) as compared to 1900–1983 (75.1 ± 29.4 Gt/year) and 1983–2003 (73.8 ± 40.5 Gt/year) [82]. These efforts confirm that we are now in an era of exceptionally rapid global ice loss.

#### Improved Mapping and Measurements of Land Ice

Technological advances continue to improve direct observations of, for example, ice flow speed [85, 86], changing gravity fields [87], and surface elevation and ice thickness [2••]. These data reveal the spatial variability of ice behavior [18], improve our understanding of seasonal patterns of ice behavior [88•], and continue to build a longer-term record of change. Spatially comprehensive datasets also provide accurate model boundary conditions. The value of consistent satellite-based glacier and ice sheet measurements is difficult to overstate, and these data play a central role in estimating mass change.

#### Understanding Changing Surface Properties and Albedo

Because surface mass balance will play an increasingly dominant role in Arctic ice loss, surface processes such as darkening have received increased focus. Recent work establishes that Greenland summertime surface albedo decreased significantly (0.02 per decade) during 1996–2012, in contrast to 1981–1996, which showed no significant albedo trend [89••]. This darkening trend is projected to continue [89••]. Likely contributors to darkening include snow grain growth, light-absorbing impurities (black carbon, organic carbon, dust), bare ice exposure, surface meltwater, and biological activity [37]. Research continues to determine the relative importance of each, now and into the future [90•].

#### Research into Firn Properties and Processes

Perennial firn aquifers, liquid water bodies in the firn, were found in southeast Greenland [91] on the heels of research highlighting the potential storage capacity of firn [92]. These studies contributed to an increased focus on the hydrological systems of glaciers and ice sheets, and subsequent research shows that firn aquifers are dynamic storage environments [93]. Runoff retention in firn, however, is limited both by pore space and by development of near-surface ice lenses, which cut off access to open pore space [94, 95•]. Early speculation that firn storage may substantially reduce near-term meltwater runoff from Greenland (including its peripheral glaciers and ice caps) seems to have been dashed by these recent findings, with a tipping point in firn capacity perhaps reached as early as 1997 [36•].

#### Improved Knowledge of Ice-Ocean Interaction

Correlation between substantial glacier retreat and increased subsurface ocean temperatures [96, 97] sparked a surge in research on the coupling between marine-terminating glaciers and the ocean. Subaqueous melt has increased since the 1990s, as a result of increased subglacial discharge and warmer subsurface ocean water [98, 99], but the impact on individual glaciers varies due to local topography, calving rate, and ice front melt rate [98, 99]. Subaqueous melt also plays a major role in mass loss for floating ice tongues. Some ice tongues remain in the Arctic, though others (e.g., Greenland’s Jakobhavn Isbræ and Petermann Glacier) have recently decayed or retreated substantially.

#### Better Observations of Subglacial Topography (and Nearshore Bathymetry)

Subglacial topography is a primary control on the rate and extent of glacier retreat in response to climate forcing. Fortunately, scientific efforts continue to measure subglacial topography and improve interpolation methods in unsampled regions [100]. For Greenland, new measurements have been incorporated into the most recent subglacial map (using mass conservation for interpolation) [2••], and this map includes estimates of near-ice bathymetry, incorporating a new method for interpolating fjord geometry [101]. Large uncertainties remain, however, in some areas of Greenland, and determining subglacial topography (and associated ice thickness) for small outlet glaciers remains problematic. Subglacial topography data for other areas of the Arctic varies in quality, and improved data continues to be a high science priority.

#### Improved Modeling of Future Changes

Researchers continue to improve models for projecting individual glacier changes. For example, several studies have used the Ice Sheet System Model (ISSM) to project changes in Greenland glaciers, including Store Gletscher in western Greenland [43] and Nioghalvfjerdsfjorden (79N) and Zacharieae Isstrøm in northeastern Greenland [44•]. These projections incorporate ocean-induced terminus melt and up-to-date subglacial topography and bathymetry, which is key to determining rates of ice loss.

#### Local/Regional Sea Level Rise Projections Based on Locations of Ice Loss

In translating land ice loss into sea level rise, the mass loss is commonly treated as total regional sums. However, the location of land ice loss (e.g., northern versus southern sectors of the GrIS) influences the location and magnitude of sea level rise. With recent improvements in projecting localized land ice loss and interpreting that loss into far-field sea level rise, local and regional sea-level estimates can be tied to the spatial partitioning of ice loss, reducing the range of predictions [78, 79•].

Together, these advances give a clearer view of ongoing Arctic ice losses, improve understanding of the processes that control ice loss rates, and aid translation of ice loss observations into actionable sea level projections for decision makers.

### Science Challenges

While estimates of total land ice mass changes consistently point to accelerating loss [11••], land ice loss continues to have the largest uncertainties among sea level rise components. Improving projections of future sea level rise from Arctic land ice loss requires advances in observations and models. These communities will have to maintain or develop new collaborations to ensure that there is a consistent feedback process in which advances on both sides help to evaluate progress and inform the direction of future research.

Observations provide the foundation for understanding the physical processes that govern ice mass loss and key boundary conditions and parameterizations for models. Improvements across these four highlight areas are key to improving process understanding and reducing uncertainties.

#### Improvement in Subglacial and Ice Thickness Data

Since even small scale (< 1 km wide) features can alter retreat rates, subglacial topography is a key dataset for projecting ice loss rates. Improved knowledge of other subglacial properties (e.g., geothermal heat flux and basal drag) and ice thickness changes across space and time are also critical for accurately modeling rates of change.

#### Better Understanding of Surface Mass Balance Controls

Understanding ice sheet and glacier hydrologic processes, including the potential for water storage, will help constrain the rate of freshwater flux to the ocean, which influences ocean properties, circulation, nutrient flux, and ocean ecosystems along with sea levels. Results that help distinguish among contributors to surface darkening will aid in projecting future surface albedo changes and associated variations in surface melt, which are also key to determining potential melt event magnitude and spatial extent.

#### Determining Calving Processes

Researchers must continue to work towards appropriate parameterizations for this multi-stage process so that it can be accurately predicted and incorporated into models in a realistic fashion [40]. This includes understanding the role of buoyancy and flexure, ice strength and structure (including surface and subglacial crevasses and zones of weakness), ice mélange and sea ice, and terminus melt.

#### Understanding Ice Sheet and Glacier Response to Varying Environmental Forcings

Given the profound changes already occurring in the Arctic and the dramatic shift expected into the future, efforts must continue to investigate the influence of a changing ocean and atmosphere on land ice. Climate states like the AMO, NAO, and atmospheric blocking events all influence ice loss [53, 102]. With increased knowledge, we can quantify variability to better characterize trends and understand the influence of potential long-term shifts in climate modes on ice loss.

As observational records continue to improve, one can envision near-term advances that combine observational records and computational techniques, such as machine learning, to make short-term projections of glacier change for both mountain glaciers and large ice-sheet outlet glaciers. Observational advances will also support improved model projections of short-term and long-term ice mass loss.

Future modeling advances can also reduce uncertainties. First, ice sheet models must incorporate a wider range of spatial and temporal scales. Higher-order ice flow models are now capable of simulating the whole GrIS on century to millennial scales [31•]. Some models use unstructured and adaptive meshes that allow lower resolution and simplified mathematics in the ice sheet interior, with higher-resolution and more computationally expensive techniques near the ice edge [103, 104]. It remains challenging, however, to resolve sub-km processes on long time scales and to capture the full range of dynamics from narrow fast-flowing glaciers to large slow-moving regions. Second, models must accurately simulate migration of the grounding line (the boundary between grounded and floating ice), which requires not only high grid resolution but also accurate bed topography [39•]. Third, models need to better represent key physical processes such as ice fracture and calving, surface darkening, englacial and subglacial hydrology, subaqueous melting, and sea ice and ice mélange buttressing.

High-resolution models with more realistic physics will enable detailed simulations of small, distinct glacier regions. For example, modeling efforts project a slow, steady retreat for Greenland’s Nioghalvfjerdsfjorden (79 North), in contrast to a rapid multi-decade retreat for Zachariae Isstrøm once it loses its floating ice tongue, with subglacial topography acting as the primary control [44•]. This type of capability needs to continue to expand towards capturing larger multi-glacier regions and, eventually, the full ice sheet. We may see Arctic-wide glacier projections within the coming decade, especially as model boundary conditions (e.g., bed topography) and parameterizations for ice-ocean interaction improve.

## Conclusions

Since the start of the century, mass loss from the Greenland Ice Sheet has accelerated. The ice sheet contributed an average of 0.8 ± 0.2 mm/year to sea level rise during 2003–2013 (out of a total global mean sea level rise of ~ 3 mm/year), with higher contributions during high-melt years such as 2012. Smaller Arctic glaciers and ice caps in Alaska, the Canadian Archipelago, the Russian Arctic, Iceland, Svalbard, and the periphery of Greenland are also losing mass, with a combined sea level contribution of about 0.5 mm/year [1••]. Surface mass balance changes, relative to changes in solid ice discharge, are more important across the Arctic and will continue to dominate into the future.

Research continues to affirm that human-caused climate change is the primary influence on Arctic (and global) ice mass loss. Until ~ 2050, most sea-level projections show little divergence across the range of RCP scenarios [74]. Planners making decisions on time scales of years to a few decades may consider sea level projections up to 2050 as a predictive basis for adaptation decisions. Beyond 2050, however, projections diverge widely. The GrIS will increasingly dominate sea level rise and mitigation choices will make a significant difference in sea level outcomes.

Decisions on action can also be aided by current progress in translating ice loss into local sea level rise projections. At least two techniques have been recently established to link local sea level changes at far field locations, like the coastal US or southern hemisphere cities, with regional ice loss from Greenland, Arctic North America, and elsewhere [78, 79•]. Such efforts are critical for providing actionable planning and management tools from scientific knowledge about ice loss. As projections of ice loss across the Arctic continue to improve, so too will decision-making information, particularly for the second half of the twenty-first century and beyond. Given that there is full agreement that ice loss will continue under all currently plausible future scenarios, planners and others are well advised not to wait to take action. Indeed, mitigation actions during this century will determine sea level rise over the next several millennia [73••], while adaptation actions can begin now to address guaranteed ice loss and sea level rise over coming decades.
